# Beneficial Immune Regulation by Biological Response Modifier Glucans in COVID-19 and Their Envisaged Potentials in the Management of Sepsis

**DOI:** 10.3389/fimmu.2022.870632

**Published:** 2022-06-27

**Authors:** Senthilkumar Preethy, Kadalraja Raghavan, Vidyasagar Devaprasad Dedeepiya, Vaddi Surya Prakash, Nobunao Ikewaki, Yasunori Ikeue, Mitsuru Nagataki, Masaru Iwasaki, Rajappa Senthilkumar, Samuel J. K. Abraham

**Affiliations:** ^1^ Fujio-Eiji Academic Terrain (FEAT), Nichi-In Centre for Regenerative Medicine (NCRM), Chennai, India; ^2^ Department of Paediatric Neurology, Sarvee Integra Private Limited, Chennai, India; ^3^ Department of Paediatric Neurology, Jesuit Antonyraj memorial Inter-disciplinary Centre for Advanced Recovery and Education (JAICARE), Madurai, India; ^4^ Mary-Yoshio Translational Hexagon (MYTH), Nichi-In Centre for Regenerative Medicine (NCRM), Chennai, India; ^5^ Department of Urology, Yashoda Hospitals, Hyderabad, India; ^6^ Department of Medical Life Science, Kyushu University of Health and Welfare, Nobeoka, Japan; ^7^ Institute of Immunology, Junsei Educational Institute, Nobeoka, Japan; ^8^ Research Division, Sophy Inc., Kochi, Japan; ^9^ Centre for Advancing Clinical Research (CACR), University of Yamanashi - School of Medicine, Chuo, Japan; ^10^ Antony-Xavier Interdisciplinary Scholastics (AXIS), GN Corporation Ltd., Kofu, Japan

**Keywords:** immune-modulation, COVID-19, biological response modifier beta-glucans, sepsis, immune-paralysis, immune cell ratios

## Abstract

Sepsis is a life-threatening condition caused by an abnormal immune response induced by infection with no approved or specific therapeutic options. We present our perspectives for the therapeutic management of sepsis through a four-way approach: (1) infection control through immune enhancement; (2) immune suppression during the initial hyper-inflammatory phase; (3) balanced immune-modulation to counter the later immune-paralysis phase; and (4) advantageous effects on metabolic and coagulation parameters throughout. COVID-19 is a virus-triggered, accelerated sepsis-like reaction that is associated with the rapid progress of an inflammatory cascade involving a cytokine storm and multiorgan failure. Here, we discuss the potential of the biological response modifiers, β-glucans (BRMGs), in the management of sepsis based on their beneficial effects on inflammatory-immune events in COVID-19 clinical studies. In COVID-19 patients, apart from metabolic regulation, BRMGs, derived from a black yeast, *Aureobasidium pullulans* strain AFO-202, have been reported to stimulate immune responses. BRMGs, produced by another strain (N-163) of *A. pullulans*, have been implicated in the beneficial regulation of inflammatory markers and immunity, namely IL-6, C-reactive protein (CRP), D-Dimer, ferritin, neutrophil-to-lymphocyte ratio (NLR), lymphocyte-to-C-reactive protein ratio (LCR), leucocyte-to-C-reactive protein ratio (LeCR), and leukocyte-to-IL-6 ratio (LeIR). Agents such as these β-glucans, which are safe as they have been widely consumed by humans for decades, have potential as adjuncts for the prevention and management of sepsis as they exert their beneficial effects across the spectrum of processes and factors involved in sepsis pathology, including, but not limited to, metabolism, infection, inflammation, immune modulation, immune enhancement, and gut microbiota.

**Graphical Abstract:**
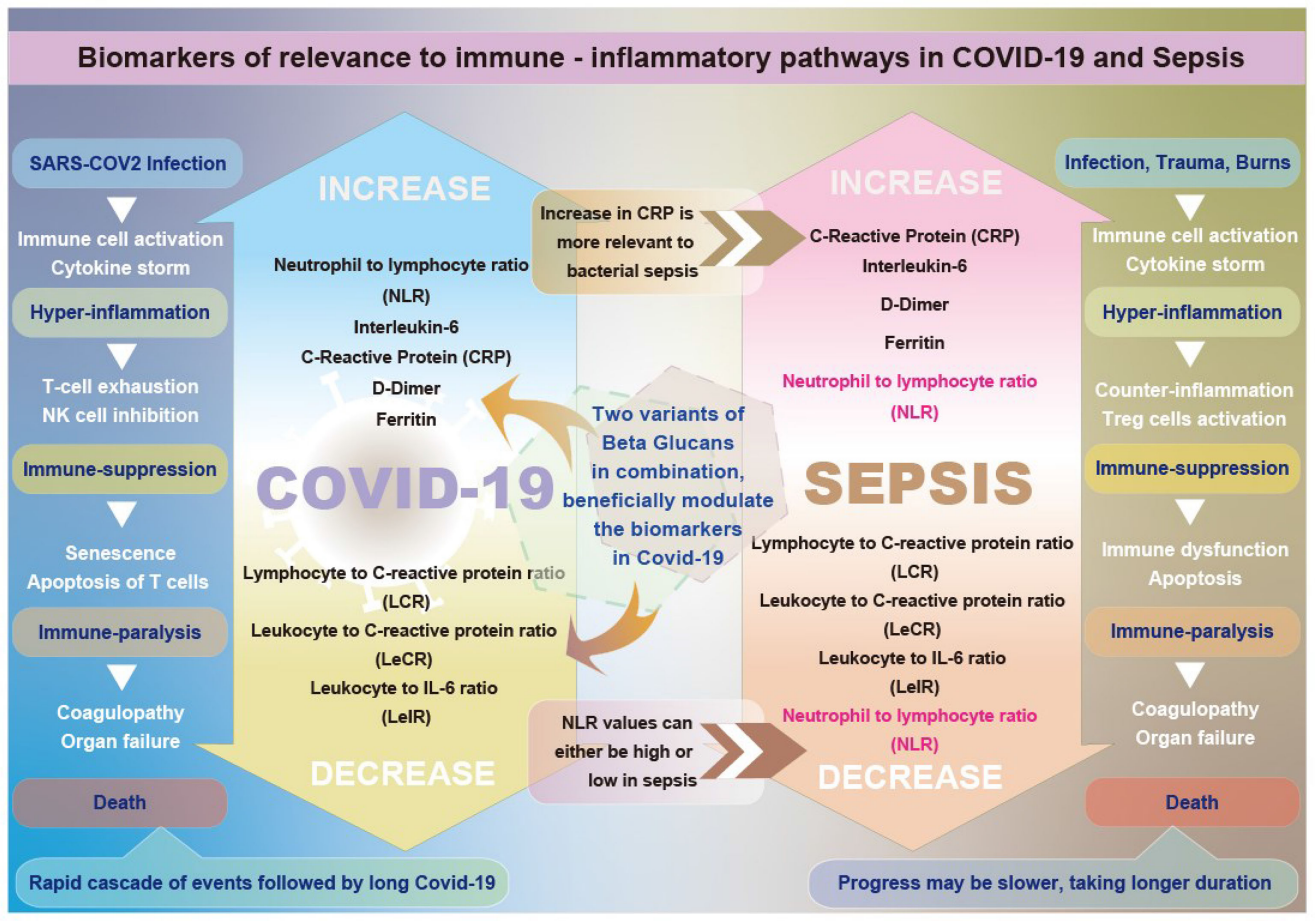
Graphical abstract illustrating the differential beneficial effects of β-glucans on biomarkers relevant to immune-inflammatory pathways in COVID-19 and sepsis.

## Background

Sepsis is a life-threatening condition caused by an abnormal immune response often induced by infection occurring in cases of severe infection, trauma, burns, shock, and major surgery. Sepsis may progress into an adult respiratory distress syndrome (ARDS), multiple organ dysfunction syndrome (MODS), and systemic inflammatory response syndrome (SIRS), leading to death (1). Globally, there has been an increase in the incidence rate of sepsis, which presents a significant societal and economic burden. Estimates suggest that in 2017, there were 48.9 million cases of sepsis, with 11 million sepsis-related deaths representing nearly 20% of all global deaths (2). Hydration, antimicrobial agents, anticoagulants, hemodynamic support, and ventilatory and respiratory support are the standard approaches to sepsis management. However, despite significant advances in medicine and healthcare, there are no sepsis-specific Food and Drug Administration (FDA, USA)-approved therapies. Instead, only supportive management approaches are used to treat sepsis. Clinical trials targeting specific pathways disrupted by sepsis have so far failed (3). When we explore the reason for such a lack of definitive or specific therapeutic options, we found that sepsis is heterogeneous, with the extent of disruption unique to each individual. A patient’s premorbid state, infection-related factors (e.g., causative organism), the site of initial infection, as well as the timing of the administered supportive therapies, are all some of the known variables leading to such heterogeneity (3). It is also important to note that infections that lead to sepsis occur in the sterile organs of the body. Therefore, a major link here is the immune system and the host–pathogen interactions (4).

Initial immune interaction involves pathogen-associated molecular patterns (PAMPs) and danger-associated molecular patterns (DAMPs) originating from bacterial or fungal organisms, as well as from the host upon injury. These PAMPS and DAMPS bind to specific pattern recognition receptors (PRRs) expressed on the surface of innate immune cells (5). This process activates proinflammatory molecules including tumor necrosis factor α (TNF-α), interleukin (IL)-1β, IL-2, IL-6, IL-8, and interferon gamma (IFN-γ), along with anti-inflammatory cytokines. This cascade leads to a variety of cellular responses and counter-responses such as enhanced phagocytic activity, vascular endothelial injury, capillary leak, synthesis of acute-phase proteins by the liver, chemotaxis of leukocytes to sites of infection/inflammation, and activation of the coagulation system. Activation of the coagulation system leads to thrombin generation, coagulation, and disseminated intravascular coagulation (DIC). The tissue factor pathway activated during the primary response causes damage to the endothelium, and the intrinsic or contact factor pathway amplifies clotting in an autoactivation manner, resulting in widespread vasodilation and the generation of bradykinin. Since these coagulation factors will be rapidly consumed, the resultant effect is diffuse hemorrhaging (5). Activation of endothelial cells, neutrophils, and monocytes also leads to the synthesis of proinflammatory mediators IL-6, IL-8, and adhesion molecules, which recruit activated leukocytes, especially neutrophils, which, in turn, produce lytic enzymes, reactive oxygen species (ROS), and nitrogen intermediates, all of which lead to microcirculatory and organ failure. The proinflammatory response was initially believed to be the cause of mortality in sepsis, and most clinical trials targeted blockade of this response by TNF and IL-1β antagonists, toll-like receptor (TLR) blockers, platelet-activating factor inhibitors, anticoagulants, and endotoxin antagonists, as well as hemofiltration to remove soluble endotoxins and cytokines, and inhibition of super-antigens. However, the results showed no benefit or, in some cases, led to worsened outcomes. This inspired an interest in the next phase of hypo-inflammatory response and immunoparalysis. Following the initial hyperinflammatory response, a cytokine storm phase—a delayed and prolonged counter-regulatory, an anti-inflammatory state is triggered. This was initially referred to as a compensatory anti-inflammatory response syndrome (CARS), later referred to as hypoinflammation (immune paralysis). Several processes lead to this immune paralysis (5). During the proinflammatory response itself, a concomitant apoptotic response occurs, leading to the depletion of T, B, and dendritic cells. The anti-inflammatory cytokine IL-10 suppresses the CD8+ T-cell response, leading to the cellular hyporesponsiveness associated with adaptive immunity. Next, the cellular exhaustion of adaptive immunity ensues, in which T lymphocytes express more inhibitory receptors, reducing their ability to produce cytokines. There is a step-wise progressive loss of T-cell functions, ending in T-cell deletion (5). As previously mentioned, the initial efforts on immune-modulating therapeutics were focused on targeting the initial proinflammatory phase. However, since that phase is relatively short-lived, drugs and molecules only had a narrow time frame to act, and phases I–III clinical trials targeting this proinflammatory cascade have had disappointing results. If the initial phase needs to be targeted, the targeting agent should be able to reduce proinflammatory cytokine production quickly. Such agents must also be able to combat the subsequent prolonged immune-paralysis phase, which leads to nosocomial infections and/or multiorgan dysfunction. Some agents that have been used to augment the host response for this phase include granulocyte colony-stimulating factor (G-CSF), granulocyte-macrophage colony-stimulating factor (GM-CSF), IFN-γ, antiprogrammed death (PD), and IL-7 (5–11). Outcomes mostly included increases in blood counts, the establishment of safety, increases in cytokine secretion by lymphocytes, enhanced human leukocyte antigen (HLA)-DR, and decreased PD-1 expression, which have all been associated with positive outcomes in the immune-paralysis phase (5–12). At present, corticosteroids remain the most widely used immune-targeting drugs for adults with sepsis (13), but they are associated with significant risks, including hypernatremia, hyperglycemia, and neuromuscular weakness (14).

With no consensus achieved so far on whether the increased mortality from sepsis is due to the infection or the immune reaction (15), there is no agent with a balanced or weighed response to an invading pathogen without compromising organ function due to immune overactivation in the early phase and a sustained immune defense in place in the later phase.

Before we move on to the focus of our perspective, we wish to also note that nutrition is another major challenge in sepsis, and the dysregulated host response induces progressive physiological alterations, leading to metabolic changes as well as impaired mitochondrial function (16).

Herein, we highlight four critical aspects of sepsis management that a single ideal sepsis-therapeutic agent must be able to address:

### Infection Control Through Immune Enhancement

In the case of antimicrobials, the main modality is the use of antibiotics for the known and often presumed pathogen(s) in sepsis. Though delayed administration of antibiotics has led to adverse outcomes, the use of broad-spectrum antibiotics is also not advocated due to the risk of antibiotic-associated adverse events and life-threatening complications due to antimicrobial resistance (17). Therefore, an ideal sepsis therapeutic agent should have a broad antimicrobial capacity with limited adverse effects. Furthermore, while these agents are effective in the initial phase, the immunosuppression phase that follows is characterized by lymphoid cell loss, leading to a diminished capacity to fight the secondary infections mediated by otherwise opportunistic organisms such as *Stenotrophomonas*, *Acinetobacter*, *Enterococcus*, *Pseudomonas*, and *Candida*. Immunomodulatory agents such as IL-7, IL-15, GM-CSF, anti-PD-1, and anti-B- and T-lymphocyte attenuator (BTLA) may modulate the host immune response, but their anti-infective or antiseptic properties remain undefined (18). Some antibiotics, such as doxycycline, have been studied for their immunomodulatory activity through inhibition of various inflammatory pathways, but they do not improve the effect of the coadministered antimicrobial agents (19). A monoclonal antibody—HA-1A—directed against the toxic lipid A component of lipopolysaccharide (LPS) was among the first developed immunomodulatory agents, but tolerance to the drug was high leading to higher mortality in the treated patients (20). Other antiendotoxin treatments such as the synthetic TLR4 antagonist were also not successful as only about half of septic patients presented with gram-negative infections (21, 22). An ideal therapeutic agent for sepsis should have additional immune-modulatory capacities apart from antimicrobial effects.

### Immune Suppression During the Initial Hyper-Inflammatory Phase

The initial trials on immunomodulatory therapies generally failed as they concentrated only on suppressing the immune response without realizing that there was an ensuing sepsis-induced immunosuppression. Anticytokine strategies were the most promising. Many of these therapies targeted TNF-α (23). While animal studies showed success, human studies showed that anti-TNF-α therapy was linked to an increased risk of infections. IL-1 receptor antagonists also showed no benefit in human clinical trials. Thus, anti-inflammatory therapies failed both due to their failure to take into account the ensuing immunosuppressive phase as well as their associated side effects (23). An ideal therapeutic agent for sepsis should modulate immune suppression wherein the immune suppression should not continue beyond the initial phase and also without the side-effects associated with the existing treatment modalities.

### Balanced Immune Modulation to Counter the Later Immune-Paralysis Phase

Immune suppression following inflammation and a failure to re-establish immune homeostasis is the major reason for mortality in sepsis. In addition, the process of trained immunity, wherein epigenetic and metabolic changes that cause persistent immune modulation and reprogramming of the patients’ immune status, accompanies the pro- and anti-inflammatory processes observed during sepsis, which further complicates the process (23). Therefore, reports suggest that it is unlikely there will be a single agent to manage the complex (24) and heterogeneous presentations of sepsis, which are highly individualized. The combination of hydrocortisone, ascorbic acid, and thiamine (HAT) has recently been advocated as an immunomodulatory treatment for patients with sepsis (25). Although the mechanism and targets of the HAT therapy have not been clarified, it is believed that ascorbic acid (vitamin C) exerts its action *via* pleiotropic immunomodulatory effects in addition to its antioxidative properties. However, a meta-analysis suggested that ascorbic acid was only effective at a dose of 3–10 g/day in critically ill patients, while lower or higher doses were not effective. While glucocorticoids (including hydrocortisone) are widely used, clinical studies have shown inconsistent results, with some showing improved outcomes while others report no or even negative effects. Macrolide clarithromycin has been under trial for its immunomodulatory properties, but the side effects need to be investigated (23). GM-CSF, a hematopoietic growth factor that stimulates the production of neutrophils, monocytes, and macrophages, has been studied in several trials, but a mortality benefit has not been observed. IFN-γ, which activates macrophages and supports the immune response against invading pathogens, has also been suggested, but there have been no randomized clinical trials. IL-7 and IL-15 are also being tried in clinical studies, but the side effects need further validation. The use of IVIg in sepsis for immune stimulation (pathogen recognition and antiapoptotic effects) and reduced inflammation (neutralizing bacterial toxins/PAMPs and inhibiting pro-inflammatory cytokine response) has been investigated for decades, but the results have been inconsistent. Immune checkpoint receptors and mesenchymal stem cells are also being tried (23). An agent capable of balanced immune modulation of reduced inflammatory cytokines and enhanced anti-inflammatory cytokines along with beneficial immune enhancement is needed.

### Advantageous Effects on Metabolic and Coagulation Parameters

Advanced age, diabetes mellitus, obesity, immunocompromised conditions such as anticancer therapies, hematological malignancies, transplantation, and other immune-impaired conditions have been reported as risk factors that converge to pose a high risk of mortality due to sepsis. Dysregulated coagulation is another important feature of sepsis. Activation of the complement system leads to cross-activation of the NLRP3 inflammasome and prothrombotic pathways. Blocking the signaling of complement C5a has shown improved survival of baboons in an *Escherichia coli*-sepsis model with lessened coagulopathy and preserved endothelial and barrier functions, but the possibility of adverse events such as coagulopathy is of concern. In addition, monoclonal Ab eculizumab, the only FDA-approved drug to block C5a, may predispose patients to meningococcal meningitis (26). An important anticoagulant pathway is the protein C system which has anti-inflammatory, antiapoptotic, and vasculoprotective effects. Administration of activated protein C (APC) did not significantly reduce mortality at 28 or 90 days, leading to the withdrawal of drotrecogin alpha from the market in 2011. Human recombinant thrombomodulin, tifacogin (recombinant tissue factor pathway inhibitor), and antithrombin III have all been tested for their ability to modulate the coagulation system but have failed to reduce sepsis mortality (23). The metabolic response that follows severe sepsis is termed “septic autocannabalism” (27). Here, there is a rapid breakdown of the body’s reserves of protein, carbohydrates, and fat. Hyperglycemia with insulin resistance, profound negative nitrogen balance, and diversion of protein from skeletal muscle to splanchnic tissues are prominent features. There are changes in cardiovascular functions with the altered flow to key metabolic sites, hypoxia, damage to the gut’s mucosal barrier, secondary organ failure, and alterations in capillary permeability. When the catabolic responses persist for more than a few days, severe malnutrition results, leading to an important risk factor for mortality. The underlying mechanism is considered to be due to inflammatory cytokines such as TNF-α, IL-1beta, IL-6, and secondary induction of catecholamines, cortisol, and glucagon by cytokines (27). Therefore, it would be ideal if the antisepsis agent could exert its benefits to counteract the other aspects of sepsis, such as metabolic dysregulation, coagulative disruption, and cardiac injury.

## Biological Response Modifier Glucans in the Four-Way Approach to the Management of Sepsis

At this juncture, where there is no single magic bullet to exert all four arms of management described above, we wish to highlight the biological response modifier beta-glucans or β-glucans (BRMGs) as a safe and wholesome immune-modulating agent for sepsis with several advantages as described below.

## Use of β-Glucans as Immunomodulators

Among the natural immunomodulators, glucans have consistently shown the highest biological effect, with more than 20,000 published studies (28). β-1,3-Glucans are highly conserved structures termed to PAMPs (27). β-Glucans can be obtained from different sources, and their biological effects are structurally dependent. Cereal-derived β-glucans contain unbranched linear chains with varying ratios of 1,3- and 1,4-glycosidic bonds, whereas fungal, lichen, and yeast β-glucans have linear backbones composed of only 1,3-glycosidic bonds and varying amounts of β-1,6-glycosidic branch points along the backbone (29). The yeast β-1,3/1,6-glucans have been shown to have better immunomodulatory effects than other types of β-glucans (28, 29). β-Glucans have been able to produce beneficial metabolic and gastrointestinal effects, altering lipid and glucose metabolism (30–32) and balancing blood glucose and cholesterol levels. Therefore, they have been consumed as supplements or adjuvants for metabolic syndrome, obesity and diet regulation, gastrointestinal conditions such as irritable bowel, and to reduce the risk of cardiovascular disease and diabetes.

## Antimicrobial Effects of β-Glucans, Specifically Yeast-Derived

β-Glucans have strong anti-infection activity, which has been shown in experimental models of infection with *Leishmania major*, *Leishmania donovani*, *Candida albicans*, *Toxoplasma gondii*, *Streptococcus suis*, *Plasmodium berghei*, *Staphylococcus aureus*, *E. coli*, *Mesocestoides corti*, *Trypanosoma cruzi*, *Eimeria vermiformis*, and *Bacillus anthracis* (29). As PAMP molecules, β-glucans are recognized by various PRRs such as Dectin-1 and CR3 (CD11b/CD18), Toll-2, lactosylceramides, and the scavenger receptor family present on the membranes of cells such as macrophages, monocytes, dendritic cells, and NK cells. β-Glucans are capable of activating the different arms of immunity. Glucan-activated B cells have been shown to secrete proinflammatory lymphokines such as IL-8 for mounting anti-infection defenses (29). β-Glucans from yeast have been reported to induce expression of a strong immune-modulatory cytokine, IL-1 receptor antagonist (IL-1Ra), and through an unknown β-glucan receptor, they are able to induce an Akt/P13K-dependant anti-inflammatory response. In a rat model of polymicrobial sepsis, β-glucan treatment attenuated proinflammatory cytokines TNF-α and IL-6 while increasing anti-inflammatory cytokine IL-10 concentrations (33). β-Glucans were able to improve immune response and survival in mice with influenza infection (30). In an *E. coli* peritoneal infection model, bacterial counts in peripheral blood reached zero in the treatment group. β-Glucans were able to enhance bacterial clearance from blood and reduce mortality in rat intra-abdominal sepsis models. By increasing circulating monocytes and neutrophils and increasing neutrophil oxidative microbicidal activity without generating harmful inflammatory responses, β-glucans enhanced the clearance of antibiotic-resistant *S. aureus* in a rat intra-abdominal infection model (30, 34). Daily dietary supplementation with β-glucan improved the vaccination response to Newcastle disease virus in chickens (35). A combination of β-glucan and vitamin C also improved healing in the treatment of infection by *M. corti* (35). Yeast-based glucans have been applied in clinical studies of bacterial and viral infections. Two independent randomized, double-blind, placebo-controlled clinical trials showed that daily oral administration of the yeast β-glucans reduced the incidence of common cold episodes during the cold season (36, 37). Another yeast-derived β-glucan was able to protect mice infected with a lethal titer of the A/Puerto Rico/8/34 (PR8; H1N1) strain of influenza virus (38). In pigs infected with swine influenza virus, administration of *S. cerevisiae* yeast β‐glucan decreased pulmonary lesion score and viral replication along with an increase in IFN‐γ and NO levels (35).

## Yeast-Derived β-Glucans in Immune Modulation

Yeast-derived β-glucans have been shown to enhance the production of IL-8 and sFas without any stimulatory effect on the production of IL-1β, IL-6, IL-12, IFN-γ, or TNF-α (39). β-Glucans have been shown to stimulate NK cell cytotoxic activity by directly binding to the NKp30 activating receptor. β-Glucans also increase the functional activity of monocytes/macrophages, and dendritic cells to stimulate the antimicrobial activity of mononuclear cells and neutrophils, accompanied by an increased proinflammatory cytokine and chemokine production and an enhanced oxidative burst. β-Glucans isolated from yeasts have been shown to have a balanced immunomodulation wherein, in addition to the above immune-enhancement effects, they have been able to enhance IL-12 and IL-10 production; modulate proinflammatory cytokine production in macrophages *via* Dectin-1/Syk signaling pathways and NLPR3 inflammasome activation; increase cytotoxic T-lymphocyte (CTL) responses; and program DC to express cell adhesion and migration mediators, antimicrobial molecules, and Th17-polarizing factors (40). β-Glucans have been shown to enhance ROS production and antibody-dependent cellular phagocytosis by neutrophils and monocytes. In clinical trials in cancer patients, β-glucans have been shown to decrease the frequency of circulating CD33^+^HLA^−^DR^−^ myeloid-derived suppressor cells (MDSCs) with improved effector function; even in advanced stages of the disease, β-glucans were able to activate the NK cells and increase the level of CD3^+^ CD56^+^ NKT and CD4^+^ CD25^+^ Treg cells (40).

## COVID-19 and Sepsis

β-Glucans can thus serve as effective immune-modulatory agents in sepsis, as they possess all four capabilities outlined above for sepsis management: mounting an anti-infection defense, immune suppression of proinflammatory cytokines, immune enhancement in the advanced immune-paralysis stage, and metabolic effects. Their potential as effective immunoadjuvants in sepsis can be observed from their effects in studies that have used them for COVID-19 (41, 42). We will explain how COVID-19 is an accelerated sepsis-like reaction before we report the outcome of the studies of β-glucans in COVID-19, making them suitable treatment adjuvants in sepsis.

The majority of studies on COVID-19 have attributed the tissue damage and multiple organ failure to the cytokine storm caused by elevated levels of proinflammatory and inflammatory cytokines such as IL-6 and IL-1β, as well as IL-2, IL-8, IL-17, G-CSF, GM-CSF, human interferon-inducible protein 10 (IP10), monocyte chemotactic protein-1 (MCP1), chemokine (C–C motif) ligand 3 (CCL3), and tumor necrosis factor (TNF), resulting in significant neutrophil and macrophage infiltration, leading to tissue damage and organ failure (43). However, recent studies have demonstrated that the increase in inflammatory cytokines in patients with severe and critical COVID-19 is actually lower than in patients with comparable conditions unrelated to COVID-19 and sepsis (e.g., acute respiratory distress syndrome (ARDS)). Furthermore, studies have shown that COVID-19-induced organ damage and death are due to immunosuppression, especially CD4+ and CD8+ T-cell lymphopenia. This lymphopenia is continuous in critically ill COVID-19 patients, leading to increased secondary infections (43–46). From this, it is clear that COVID-19 closely resembles sepsis. Similar to sepsis, immune suppression and immune paralysis in the later phases cause tissue damage and mortality in COVID-19.

Therefore, COVID-19 likely represents a fast-track version of sepsis (47) with the immune paralysis that follows, leading to aftermath effects and the long-COVID syndrome (48).

## Use of *A. pullulans*-Derived β-Glucans as Immunomodulators Against COVID-19

**Figure 1:**
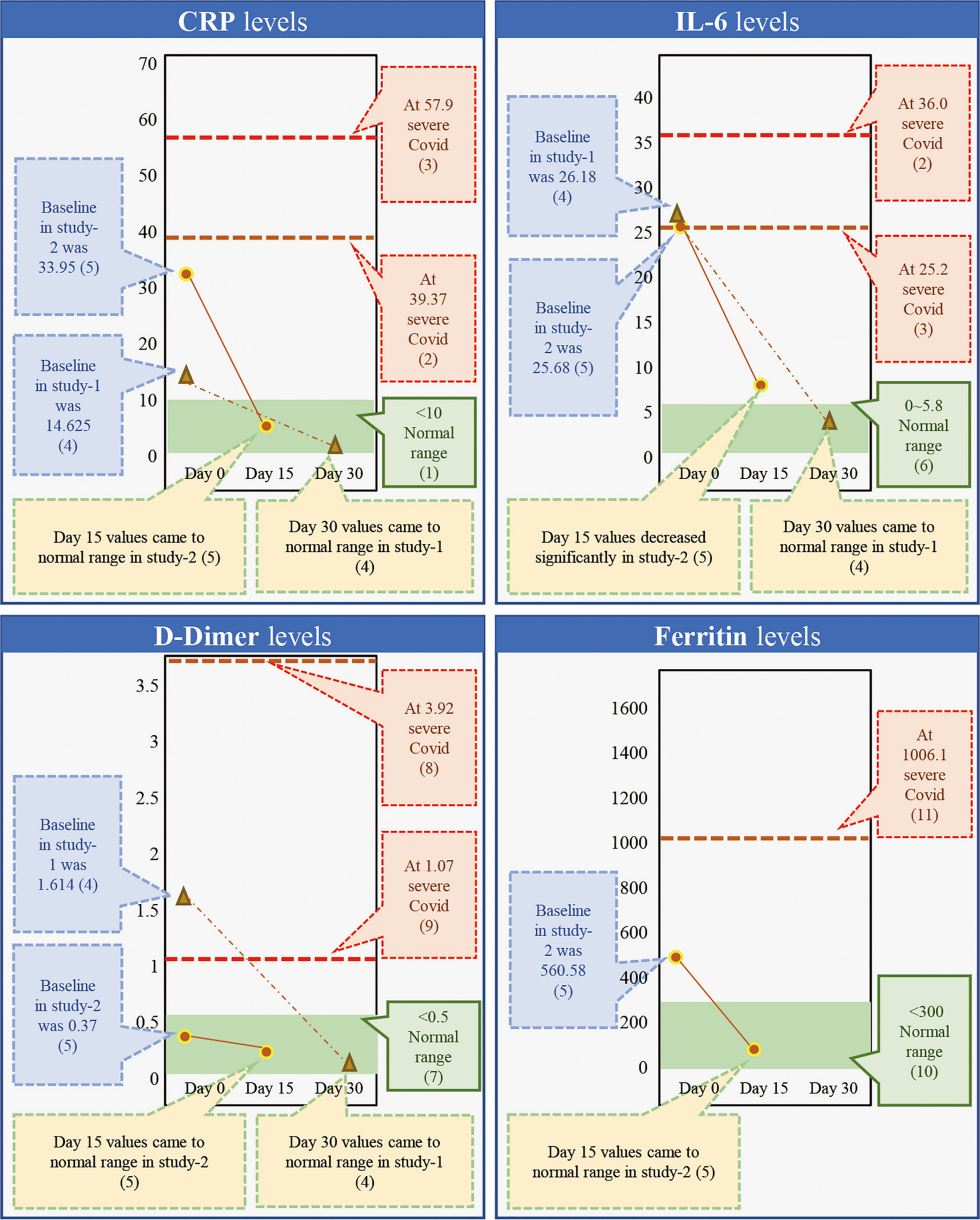
Comparison of markers of inflammation, coagulation, and immunity (CRP, IL-6, D-dimer, and ferritin) associated with severe COVID-19 with a high risk of mortality from the literature against the outcome of studies done on consumption of *Aureobasidium pullulans* strain (AFO-202 and N-163)-derived β-glucans in COVID-19 patients demonstrates that the marker levels were regulated to the normal range after 15 days of consumption and the values remained in the normal range post-30 days as well [(1) Ref. no (56).; (2) Ref. no (54).; (3) Ref. no (55).; (4) Ref. no (41).; (5) Ref. no (42).; (6) Ref. no (57). (7) Ref. no (58).; (8) Ref. no (59).; (9) Ref. no (60).; (10) Ref no (61).; (11) Ref no (62).].

**Figure 2:**
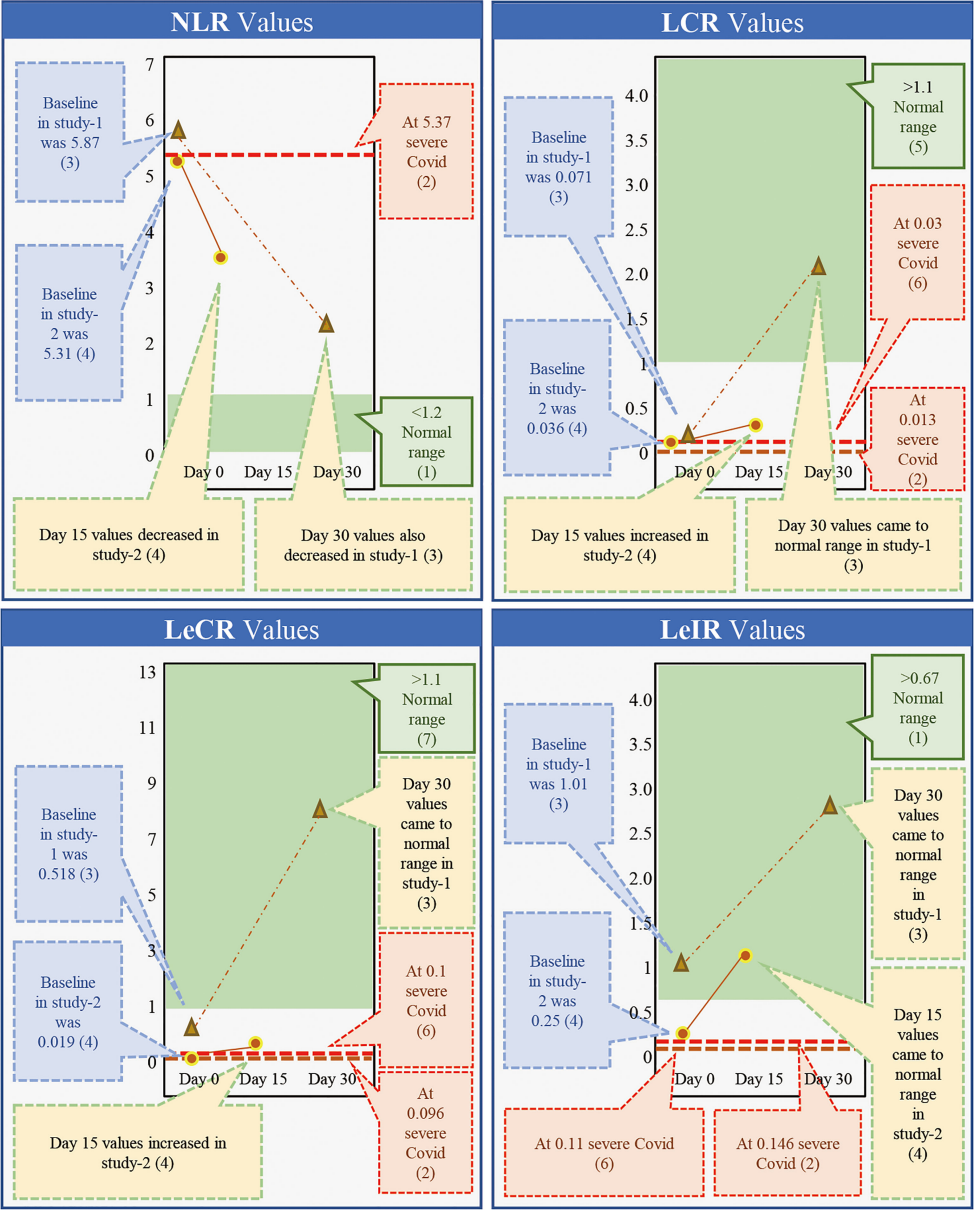
Comparison of markers of immunity (NLR, LCR, LecR, and LeIR) associated with severe COVID-19 with high a risk of mortality from the literature against the outcome of studies done on consumption of *Aureobasidium pullulans* strain (AFO-202 and N-163)-produced β-glucans in COVID-19 patients demonstrating that the marker levels were regulated to normal range after 15 days of consumption and the values remained in the normal range post-30 days as well [(1) Ref. no (66).; (2) Ref. no (55).; (3) Ref. no (41).; (4) Ref. no (42).; (5) Ref. no (63).; (6) Ref. no (55).; (7) Ref. no (64).].

While β-glucans of different chemical compositions are available, their processing for extraction and purification varies based on their functional properties. In addition, their biological response-modifying actions also vary (29). We have previously shown that *A. pullulans*-derived β-glucans are secreted by black yeast as an exopolysaccharide, thereby negating isolation or extraction methods (41, 42, 49). Two different strains of *A. pullulans*, AFO-202 and N-163, produce β-glucan as an exo-polysaccharide with the same chemical composition (C6H10O5)*n* but a different structural formula. The difference in the structure leads to functional differences. The AFO-202 β-glucan has beneficial effects on glucose metabolism and immune activation by stimulating the production of IL-8 and sFAS while suppressing inflammatory cytokines such as IL-1β, IL-2, IL-6, IL-12 (p70+40), IFN-γ, TNF-α, or soluble Fas ligand (sFasL) (50, 51). In comparison, N-163 has positive effects on lipid metabolism and more potent anti-inflammatory effects, as well as antifibrotic effects and beneficial immunomodulation of coagulation-associated and anti-inflammatory markers associated with a decrease in CD11b, serum ferritin, galectin-3, and fibrinogen. Even among yeast-derived β-1,3-1,6-glucans, the structure of *A. pullulans* β-glucans is unique for β-glucans, and its purity adds to its superior beneficial effects (29). Despite the disadvantage of purification in the process of production, water solubility remains another major hurdle, preventing the easy clinical administration of β-glucans (50–53). However, *A. pullulans*-derived β-glucans are water-soluble with higher purity and functionality, making oral administration possible. We have previously conducted two human clinical studies to study the effects of β-glucans in COVID-19 (41, 42). Here, we observed a continuous and sustained decrease in erythrocyte sedimentation rate (ESR), D-Dimer, IL-6, and ferritin expression for up to 30 days. In the control groups, there was an initial decrease followed by an increase in the expression of these molecules. Effective immune enhancement and modulation were observed as a decrease in the NLR with an increase in the lymphocyte-to-CRP ratio (LCR) and leucocyte-to-C-reactive protein ratio (LeCR) (41, 42). In a study conducted on healthy volunteers, these β-glucans were shown to exert a wholesome, balanced immunomodulation wherein there was metabolic control of glucose evident as a decrease in HbA1C and glycated albumin (GA). There was also an immune enhancement with a significant increase in eosinophils and monocytes and a marginal decrease in D-dimer levels. Along with this, there was a decrease in the NLR and an increase in the LCR and LeCR. In addition, we observed some regulation of lipids due to a decrease in total and LDL cholesterol. We also observed immunomodulation of coagulation-associated and anti-inflammatory markers through a decrease in CD11b, serum ferritin, galectin-3, and fibrinogen (49). When we conducted a literature search to understand the effects of AFO-202- and N-163-derived β-glucans on key inflammatory and immune markers, we found one relevant study that was carried out for 30 days (41) and another 15-day study (42) in patients with COVID-19. Here, the authors reported that the C-reactive protein (CRP) at levels of 57.9 and 39.37 mg/L caused severe COVID with a high risk of mortality (54, 55). While reported in the literature, in the studies of *A. pullulans-*derived β-glucans (41, 42), the baseline values of 33.95 and 14.62 mg/L returned to normal ranges (56) within 15 and 30 days, as well (38, 39) (**Figure 1**). Moreover, IL-6 at levels of 36.0 and 25.2 pg/mL (57, 58) has been associated with severe COVID and a high risk of mortality. In studies of *A. pullulans*-derived β-glucans, baseline values of 26.18 and 25.68 pg/ml decreased to normal ranges (59) in both 15- and 30-day durations (38, 39) (**Figure 1**). With D-dimer levels of 3.92 and 1.07 μg/ml (60, 61), severe COVID with a high risk of mortality was reported in the literature. In the studies of *A. pullulans*-derived β-glucans, baseline values of 1.614 and 0.37 μg/ml decreased to normal ranges (62) after 15 and 30 days (41, 42) (**Figure 1**). In ferritin levels associated with severe COVID being 1,006.1 ng/ml (61), the baseline value of 560.58 ng/ml returned to normal (63) in 15 days (41). NLR values associated with severe COVID and mortality were 5.37 (64), but decreased significantly in *A. pullulans*-derived β-glucan studies (41, 42). In reports from the literature (55, 65), LCR values also decreased in severe COVID-19 (0.03, 0.13) and increased to normal values after *A. pullulans*-derived β-glucan consumption (**Figure 2**). LeCR and LeIR, which were not well studied earlier, have been reported as important markers for COVID-19-associated sepsis and mortality (57). LeCR values of 0.1 and 0.096 reported in severe COVID were 0.019 and 0.518 (54, 55) at baseline in the *A. pullulans*-derived β-glucans, which increased to normal values (57) in 30 days (**Figure 2**). Similarly, LeIR also increased to a normal range (57) in studies of both 15 and 30 days (41, 42) (**Figure 2**). The advantageous effects on LeCR, LCR, and NCR (41, 42) present an opportunity to use this glucan as a biomarker in assessing the immune parameters of other β-glucans and immune-enhancing and/or immune-modulating food supplements (52, 53). In addition, a rapidly responding immune-modulating capability during COVID-19 infection against a rapid cytokine storm (41, 42) simultaneously enhances immune defense and resolves dysfunctions in coagulability (67) with the combination of two variants.

## Use of β-Glucans in the Four-Way Management of Sepsis With Emphasis on *A. pullulans*-Derived β-Glucans

In the COVID-19 studies of the *A. pullulans* β-glucans (41, 42) described above, apart from the decrease to the normal range, there is sustained maintenance of the levels past the acute phase, establishing the potential of these β-glucans for the management of the immune paralysis or postacute inflammatory phase that occurs in sepsis. It is important to note that D-dimer and ferritin have already been suggested as important prognostic markers for evaluating treatment efficacy and monitoring septic patients for long periods of time (68–70).

Another crucial factor to be considered in sepsis management is the gut microbiome, whose disruption is a potential risk factor for sepsis and subsequent organ dysfunction. Several studies have established a correlation between gut microbiome changes or dysbiosis and sepsis risk and progression (71). Herein, β-glucans become useful in this context; when taken as supplements, the main mechanism by which they exert immune modulation effects is through their action on the reconstitution of the gut microbiome. The *A. pullulans*-derived β-glucans have been shown to result in a beneficial reconstitution of gut microbiota and control of α-synuclein and curli-amyloid-producing enterobacteria (72). This is significant because enterobacteria-produced endotoxins have been known to play a major role in the pathophysiology of sepsis and its complications (73). However, before proceeding to consider these results for sepsis management, further parameters need to be evaluated, including (1) the effect against evolving strains of viruses and bacteria, (2) the effects of β-glucans on HLA-DR and individual HLA haplotypes, and (3) the effects on gut microbiota in clinically relevant models of sepsis, to name a few. Nevertheless, the valuable effects of β-glucans described above make them potentially promising therapeutic immunoadjuvant agents for sepsis given their established safety by human consumption over decades, capabilities of mounting a four-way approach to sepsis management, anti-infection defense, immune suppression of proinflammatory cytokines, immune enhancement in the advanced immune-paralysis stage, and metabolic effects after validation by translational and clinical studies. However, **Table 1** shows the four-way approach to interventions in sepsis and COVID-19 and the relevance of A. pullulans β-glucans whose effects cover the effects produced by the other interventions. **Table 2** shows the immunomodulatory actions of the some of the β-glucans.

**Table 1:** Four-way approach to interventions in sepsis, COVID-19, and the relevance of *A. pullulans* β-glucans whose effects cover the effects produced by the other interventions.

Four-way approach to management of Sepsis and COVID-19	Intervention	Sepsis	Effects		COVID-19	Effects		Relevance/Effects of *A.pullulans* beta glucans
			Increase	Decrease		Increase	Decrease	
**Anti-microbial**	Antibiotics			Infection			Infection	Anti-viral and antibacterial (39, 74)
**Immune-suppression**	Corticosteroids	Ref (75)		NF-κB, IL-1, TNF, IL-8, and MCP-1	Ref (76)		Cytokines especially IL-6	NF-κB downregulation; Suppression of NO, IL-1β, IL6, TNF-α (77)
	C5a blockade (78)	Yes		IL-17				Suppression of inflammatory cytokines (50)
	Anakinra (IL-1 receptor antagonist)	Ref (79)			Ref (79)		CRP, IL-1	Decrease in CRP and IL-1 (41, 42)
	Tocilizumab and sarilumab (IL-6 blockade)	Ref (80)		IL-6	Ref (81)		IL-6	Decrease in IL-6
**Immune-enhancement**	IFN-γ,	Ref (82)	HLA-DR expression ; Th1 cytokine					Th1 cytokine increase (83)
	GM-CSF	Ref (84)	Proinflammatory monocytic cytokine production		Both administration and inhibition of GM-CSF (85)	Administration of GM-CSF: Alveolar macrophages (85)	Inhibition of GM-CSF :IL-1, IL-6, TNF (85)	Cytokine increase, increase in immune cells such as NK cells (38, 86); Increase in GM-CSF expression (39)
	Interleukin-7	Ref (10)	CD4+ and CD8+ T cells					Increase in CD4+ and CD8 lymphocytes (41, 87)
	Intravenous Immunoglobulins	Ref (88)		IL-1,IL-6, IL-10,IL2,TNF-α	Ref (89)	IL-13, CD4+ T Cells	IL-6	Decrease in IL-1β, IL-2, IL-6, IL-12 (p70+40), IFN-γ, TNF-α) (38); Increase in IgA (90)
**Metabolic/Coagulation**	Metabolic resuscitators [coenzyme Q10 (CoQ10), cytochrome oxidase (CytOx), L-carnitine, melatonin]	Ref (91)	Mitochondrial function					Improvement in glucose, lipid levels (31, 32) and mitochondrial respiration (92)
	Ascorbic acid, tocopherol, selenium and zinc	Ref (91)	Mitochondrial function					
	Ascorbic acid and Zinc					NK cells, T cells	Neutrophils	Increase in NK cells, T cells and decrease in neutrophils (38, 41, 42, 86)
	Anti-coagulation therapies	Ref (93)		Ferritin, DDimer				Decrease in Ferritin ad D-Dimer (41, 42)

**Table 2:** Immunomodulatory actions of different β-glucans.

S. No	β-Glucans Source/Type	Model/Organism	Immuno-modulatory effects	Reference
1	Plant derived (Sparassis crispa, Phellinus linteus, Platycodon grandiflorum, Cordyceps millitaris, and Angelica gigas Nakai)	*In vitro*	DC maturation; Translocation of NF-κB subunits to the nucleus	(94)
2	Mushroom and barley	*In vitro*	PBMC proliferation; IL-12 and IL-10 production	(95)
3	Cellulose	*In vitro*	No immunomodulation	(95)
4	Spent Brewers’ Yeast (Saccharomyces cerevisiae)	In vivo - rats and human clinical trials	Phagocytosis, oxidative burst, production of cytokines and chemokines in dendritic cells and macrophages; interleukin-1β (IL-1β)	(96)
5	Curdlan (Alcaligenes faecalis)	*In vitro*	Activation of MAPKs and NF-κB Pathways; M1 Phenotype Polarization	(97)
6	Euglena gracilis	*In vitro* and animal studies	Transactivation of NF-κB; expression of proinflammatory mediators (TNF-α, IL-6, COX2, and iNOS); production of high level of NO	(98)
7	Synthetic Beta-glucan (βglu6)	*In vitro*	Secretion of large levels of cytokines and chemokines, including CD54, IL-1α, IL-1β, IL-16, IL-17, IL-23, IFN-γ, CCL1, CCL3, CCL4, CCL12, CXCL10, tissue inhibitor of metalloproteinase-1 (TIMP-1) and G-CSF in murine macrophages as well as IL-6, CCL2, CCL3, CCL5, CXCL1 and macrophage migration inhibitory factor (MIF)	(99)
8	Mushroom Pleurotus ostreatus	Human clinical trials	Increased number of circulating natural killer cells as well as a preventive effect on the reduction of natural killer cell activity	(100)
9	PGG Glucan Saccharomyces cerevisiae	*In vitro*	Enhanced neutrophil anti-microbial functions	(101)
10	Fungus Sclerotinia sclerotiorum IFO 939	*In vitro*	Enhanced Phagocytic activity and interleukin-1 (IL-1) production	(102)
11	Shiitake medicinal mushroom, Lentinus edodes (Berk.) singer mycelium	Human clinical trial	Increase in the number of circulating B-cells	(103)
12	Algae	*In vitro*	Increase in phagocytic activity; stimulation of IL-2 secretion	(104)
13	Agrobacterium-derived			
14	Oats			
15	Pustulan (β-(1,6)-glucan), lichenan (β-(1,3)-(1,4)- glucan), xyloglucan (β-(1,4)- glucan), and pullulan (α-(1,4)- (1,6)-glucan)	*In vitro*	Strong cytokine production	(105)
16	Aureobasidium pullulans	*In vitro*, animal models and human clinical studies	Increase in production of interleukin-8 (IL-8) or soluble Fas (sFas); Decrease in IL-1beta, IL-2, IL-6, IL-12 (p70+40), interferongamma (IFN-gamma), tumor necrosis factoralpha (TNF-alpha) or soluble Fas ligand (sFasL); Decrease of CRP, Ferritin and DDimer; Increase in NK cells and T cells; Activation of Dendritic cells; Activation of MAPKs and NF-κB Pathways; Stimulation of Macrophages	(38, 39, 41, 42, 49–51)

## Concluding Remarks

Further research into the effects of these two variants, independently and together, at different proportions on each type of immune response or cell or biological process may make this a future biomarker for evaluating biological systems. Unlike other β-glucans, the availability of two different *A. pullulans*-derived β-glucans that have been proven to be safe and play a complementary role in yielding health benefits to humans is likely to have several hidden potentials. This potential needs to be further studied with additional research, especially with reference to their beneficial effects on the gut microbiome (68, 72, 73) and their derivative metabolomes. This research is ongoing in both preclinical and clinical studies, making glucans effective not only in the management of sepsis but also as prophylaxis in immune-compromised individuals who are at high risk of developing sepsis during critical infections. With inflammation and infection being two major arms of the balance governing the pathogenesis of sepsis confounded by a complex immune system reaction occurring at a swift pace, the biological response-modifying agents such as the β-glucans, whose safety has been proven, may be potential adjuncts throughout treatment as they exert their beneficial influence across the spectrum of processes and factors occurring in sepsis, including, but not limited to, metabolism, infection, inflammation, immune modulation, immune enhancement, and the gut microbiota.

Further studies will make it evident whether people who have been consuming these β-glucans over the years, when challenged by infection, inflammation, or autoimmune diseases, have a lower risk of progression to the critical stage of sepsis or if sepsis develops, if their prognosis is better. This will then make BRMGs highly suitable for prophylaxis (106) as well. The prophylaxis concept gains significance when considering the potential biosafety threat of viruses such as NeoCoV (107), which uses an angiotensin-converting enzyme 2 (ACE2) for entry into humans similar to the SARS-CoV2 virus, as the implications of immune-modulation cover sepsis and sepsis-like reactions that occur in viral infections such as COVID-19. Since immunomodulation is the prime target in combatting sepsis, the severity and risk of mortality can be better monitored by immune markers such as NLR, LCR, LeCR, and LeIR (41, 42). Furthermore, β-glucans have been reported to mitigate disease severity by interaction with ACE-2 receptors (108) as well as by modulating immunity to normalize NLR, LCR, LeCR, and LeIR expression (41, 42). These nutritional supplements may serve as effective prophylactic options to avoid a catastrophic future pandemic and novel sepsis-like reactions that may accompany them when consumed regularly.

## Author Contributions

SA and KR contributed to the conception and design of the study. RS helped in the literature search. SA and SP drafted the manuscript. VD, NI, YI, MN, and MI performed critical revisions to the manuscript. All the authors read and approved the submitted version.

## Conflict of Interest

Author SA is a shareholder in GN Corporation., Kofu, Japan which in turn is a shareholder in the manufacturing company of novel betaglucans using different strains of Aureobasidium pullulans. MN and YI are employees of Sophy Inc., Kochi, Japan, manufacturers of novel betaglucans using different strains of Aureobasidium pullulans. Author KR was employed by Sarvee Integra Private Limited, Chennai, India.

The remaining authors declare that the research was conducted in the absence of any commercial or financial relationships that could be construed as a potential conflict of interest.

## Publisher’s Note

All claims expressed in this article are solely those of the authors and do not necessarily represent those of their affiliated organizations, or those of the publisher, the editors and the reviewers. Any product that may be evaluated in this article, or claim that may be made by its manufacturer, is not guaranteed or endorsed by the publisher.
